# Effects of Bile Acids and the Bile Acid Receptor FXR Agonist on the Respiratory Rhythm in the *In Vitro* Brainstem Medulla Slice of Neonatal Sprague-Dawley Rats

**DOI:** 10.1371/journal.pone.0112212

**Published:** 2014-11-18

**Authors:** Cong Zhao, Xianbao Wang, Yuling Cong, Yi Deng, Yijun Xu, Aihua Chen, Yanru Yin

**Affiliations:** 1 Department of Cardiology, Zhujiang Hospital of Southern Medical University, Guangzhou, China; 2 Joint Surgery, Central Hospital of Shengli Oil Field, Dongying, China; 3 Department of Physiology, Southern Medical University, Guangzhou, China; Nihon University School of Medicine, Japan

## Abstract

Intrahepatic cholestasis of pregnancy is always accompanied by adverse fetal outcomes such as malfunctions of respiration. Farnesoid X receptor (FXR) plays a critical role in the homeostasis of bile acids. Thus, we are determined to explore the effects of farnesoid X receptor (FXR) and five bile acids on respiratory rhythm generation and modulation of neonatal rats. Spontaneous periodic respiratory-related rhythmical discharge activity (RRDA) was recorded from hypoglossal nerves during the perfusion of modified Krebs solution. Group 1–6 was each given GW4064 and five bile acids of chenodeoxycholic acid (CDCA), deoxycholic acid (DCA), lithocholic acid (LCA), cholic acid (CA) as well as ursodeoxycholic acid (UDCA) at different concentrations to identify their specific functions on respiratory rhythm modulations. Group 7 was applied to receive FXR blocker Z-guggulsterone and Z-guggulsterone with the above bile acids separately to explore the role of FXR in the respiratory rhythm modulation. Group 8 was given dimethyl sulfoxide (DMSO) as controls. Apart from UDCA, CDCA, DCA LCA and CA all exerted effects on RRDA recorded from hypoglossal nerves in a concentration-dependent manner. Respiratory cycle (RC), Inspiratory time (TI), Expiratory Time (TE) and Integral Amplitude (IA) were influenced and such effects could be reversed by Z-guggulsterone. FXR may contribute to the effects on the modulation of respiratory rhythm exerted by bile acids.

## Introduction

Conversion of cholesterol to bile acids plays an important role in the cholesterol homeostasis and serves as the main pathway for cholesterol catabolism [1]. Bile acids are normally produced in the liver cells and secreted into the intestines. The regular enterohepatic circulation of bile acids is essential for the processing and absorption of dietary fat [2], [3]. Bile acids are also known as signaling molecules that activate the farnesoid X receptor (FXR) [4].

As one of the members in the nuclear hormone receptor superfamily, FXR is highly expressed in liver, kidney, adrenal glands and intestine. As the target genes of FXR like bile salt export pump (BSEP) and small heterodimer partner (SHP), FXR is able to maintain the homeostasis of cholesterol and bile acids via regulating their expressions by binding to the FXR response element (FXRE) located on the promoters of these genes [5]. FXR can be activated by primary and secondary bile acids including chenodeoxycholic acid (CDCA), deoxycholic acid (DCA), lithocholic acid (LCA) and cholic acid (CA) but with decreasing potency [6]. However, synthetic FXR agonists such as GW4064 which is highly specific for FXR, has made it possible for FXR to become the potential therapeutic targets in curing diseases [7], [8]. FXR antagonists such as guggulsterone are able to stimulate the conversion of cholesterol to bile acids. Guggulsterone is reported to inhibit FXR induction of ileal bile acid binding protein (IBABP) and SHP expression and is used to lower serum levels of cholesterol and triglyceride [9]. Taking the abilities of inhibiting FXR-mediated effects into consideration, FXR antagonists may have therapeutic potential.

However, excessive serum concentrations of bile acids are toxic to the cells. As a multifactorial liver disorder in clinical scenarios, intrahepatic cholestasis of pregnancy is associated with abnormal bile acid levels in serum, causing not only maternal itching but also high infant morbidity and mortality [10]. Inhalation of biliary reflux in the lungs will cause acute and chronic dyspnea and in the healthy fetuses, inhalation of amniotic fluids combined with meconium will be likely to cause pulmonary atelectasis [11], [12]. Elevated bile acids in the serum may induce abnormalities of pulmonary surfactant system and thereby are involved in the pathogenesis of respiratory distress syndrome [13]. In pregnant rats, the bile acid accumulation in the fetal lung caused by cholestasis is likely to result in lung macrophages infiltration and inflammatory response [14]. A recent study has proved that intrahepatic cholestasis of pregnancy can be modulated by promoter DNA methylation in nuclear receptors such as farnesoid X receptor (FXR) and pregnane X receptor (PXR) [15]. Thus, we speculate that FXR may be involved in respiratory disorders which are mostly observed after inhalation of biliary reflux in the lungs.

Rhythmic breathing is essential for blood oxygenation and survival. Respiratory movements shall be spontaneously produced from birth onwards. Within the brainstem, a complex neural network plays a critical role in periodic respiratory generation. Numerous studies show that the respiratory rhythm seems to be generated in the medulla, whereas the exact place of respiratory rhythm generation remains controversial [16]–[19]. It is likely that the medial region of the nucleus retrofacialis (mNRF) is responsible for the periodic respiratory rhythm generation. However, there are also researches indicating that the pre-Bötzinger complex (PBC) could generate eupneic rhythmicity [16], [20]–[22].

Our previous study showed that there was a significant change in the respiratory function in animals with high serum concentration of bile acids. Bile acids in the serum can have direct effects on respiratory functions besides reflex responses. Still, the underlying mechanisms remain debatable [23]. So, this research aims to study the influence of five bile acids and FXR blocker Z-guggulsterone on the periodic respiratory-related rhythmical discharge activity (RRDA) recorded from hypoglossal nerves of brainstem slice of neonatal rats. This study provides evidence on the regulatory role of bile acids on periodic respiratory rhythm generation and modulation through FXR, further proving the involvement of FXR in the respiratory functions.

## Methods

### Ethics Statement and Animals

Neonatal Sprague-Dawley rats (0-3d) were obtained from the Laboratory Animal Center of Southern Medical University (SCXK 2006-0015). All the experimental procedures were in compliance with the National Institutes of Health Guidelines for Care and Use of Laboratory Animals and received approval from the Bioethics Committee of Southern Medical University.

### Reagents

Z-guggulsterone was purchased from Santa Cruz Biotechnology (Santa Cruz, CA, USA), Chenodeoxycholic acid (CDCA), deoxycholic acid (DCA), ursodeoxycholic acid (UDCA) were purchased from Shanghai Yiji Co., Ltd (Shanghai, China). Lithocholic acid (LCA) and cholic acid (CA) were purchased from Shanghai Guidechem Co., Ltd (Shanghai, China).

### Brainstem Medulla Slice

Under deep ether anaesthesia, the head and vertebral column of Sprague-Dawley rats from birth to 3 days of age were isolated and placed in a dissection chamber filled with the modified Krebs solution (NaCl, 124 mmol/L; KCI, 5.0 mmol/L; KH2PO4, 1.2 mmol/L; CaCl_2_, 2.4 mmol/L; MgSO_4_, 1.3 mmol/L; NaHCO_3_, 26 mmol/L; glucose, 30 mmol/L). The cerebrum was quickly removed as well as the cerebellum by transacting at the intercollicular level. The above procedures were completed within 2–3 min. The cranial and the spinal nerves were retained as much as possible. The preparation which included the medial region of the nucleus retrofacialis (mNRF), the pre-Bötzinger complex and part of the ventral and dorsal respiratory group with the hypoglossal nerve roots was then placed in a 0.5–2 ml bath and perfused at a rate of 4–5 ml/min with modified Krebs solution (pH 7.35–7.45) equilibrated with 95% O_2_ – % CO_2_ gas. The temperature of the perfusion medium was kept at 27°C [24].

### Recordings

In order to achieve extracellular recording from hypoglossal nerves, tightly fitting glass capillary suction electrodes (inner diameter of the tip 50–130 µm) were used. Filled with modified Krebs solution, the electrodes were connected through Ag-AgCl wires to pre-amplifiers. Spontaneous periodic respiratory-related rhythmical discharge activity (RRDA) was recorded from hypoglossal nerves and displayed on the screen. The output of the pre-amplifier was fed to a recorder. Respiratory cycle (RC), inspiratory time (TI), expiratory time (TE) and integral amplitude (IA) of respiratory-related rhythmical discharge activity (RRDA) were then analyzed. Drugs were dissolved in the modified Krebs solution and applied by perfusion [24].

### Experimental Protocol

This experiment was divided into 8 groups: Group 1 was conducted to observe the effects of GW4064 (3 µM) on RRDA recorded from hypoglossal nerves. Group 2–6 was each conducted to observe the effects of five different bile acids (CDCA, DCA, LCA, CA and UDCA) on respiratory rhythm generation and modulation. The above five bile acids were dissolved in dimethyl sulfoxide (DMSO) at different concentrations (10^−1^∼10^−5^ mg/mL CDCA, 10^−1^∼10^−5^ mg/mL DCA, 1∼10^−4^ mg/mL LCA, 1∼10^−3^ mg/mL CA and 1∼10^−3^ mg/mL UDCA). After RRDA was stably recorded from hypoglossal nerves for at least 10 minutes during which data recorded was used as controls, the bile acids at different concentrations were then applied. The bile acids were washed out after experiments were finished. Group 7 was conducted to observe the effects of FXR blocker Z-guggulsterone (20 µM) and combination of Z-guggulsterone and the above four bile acids (CDCA, CA, DCA and LCA). After RRDA was stably recorded for at least 10 min during which the data recorded was used as controls, Z-guggulsterone was firstly applied. After 15 minutes of observation, Z-guggulsterone was washed out. The combination of Z-guggulsterone and the above four bile acids was then applied after full recovery of RRDA. Group 8 was conducted with DMSO (final concentration was 0.5%) as control. As the time when the effects of bile acids or other agents took place varied in each experiment, we waited for about 10min after the effects first showed up to make sure our results were stable and convincible and recorded the data for at least 15min during which the middle 10min of data was used to analyze.

### Bile Acids Treatments

Intraperitoneal injection of bile acids (CDCA, CA, DCA, LCA and UDCA at 50 mg/kg) and GW4064 (50 mg/kg) were performed on neonatal Sprague-Dawley rats (0-3d). The injections were conducted 6 times with an interval of 40 min. The brains were separated 30min after the last injection and were prepared for RT-PCR.

### Real-time reverse transcription-PCR (RT-qPCR)

Total RNA was isolated from tissue samples with Trizol (Invitrogen) according to the manufacturer's instructions. RNA was reverse transcribed by M-MLV Reverse Transcriptase (Promega, WI, USA) according to the manufacturer's protocol. Sequence-specific primers for FXR and SHP were, respectively, shown in *Table 1*.

**Table 1:** Sequence-specific primers for FXR and SHP.

Rat-GAPDH-F	GCAAGAGAGAGGCCCTCAG
Rat-GAPDH-R	TGTGAGGGAGATGCTCAGTG
Rat-FXR-F	CCAGGTCTCACTCAAGAATC
Rat-FXR-R	GTCCCTAGCTCAGTTGACAA
Rat-SHP-F	ATCCTCCTCATGGCTTCCAC
Rat-SHP-R	CCCATTCTACAGGTCACCTC

Real-time PCR was performed with qPCR Master Mix (Promega, WI, USA) on a Real-Time PCR detection instrument using the SyBr Green detection protocol as outlined by the manufacturer. Briefly, the amplification mixture consisted of 0.5 µM primers, 10 µl of qPCR Master Mix, and 1.5 µl template DNA in a total volume of 20 µl. Samples were amplified with the following program: initial denaturation at 95°C for 30 sec, followed by 40 cycles of denaturation for 15 s at 95°C and annealing/elongation for 60 s at 60°C. All PCRs were run in triplicate, and control reactions without template were included.

### Statistics

All experiment data was processed by SPSS 17.0. All experiment results were shown using sample mean ± standard deviation and student's t tests were used to test statistical significance. For comparisons in the RT-PCR test, data were analyzed using a one-way analysis of variance (ANOVA), followed by LSD test. P<0.05 indicates a significant difference.

## Results

### 1. Effects of Bile Acids and GW4064 on FXR-SHP Pathway

**Figure 1:**
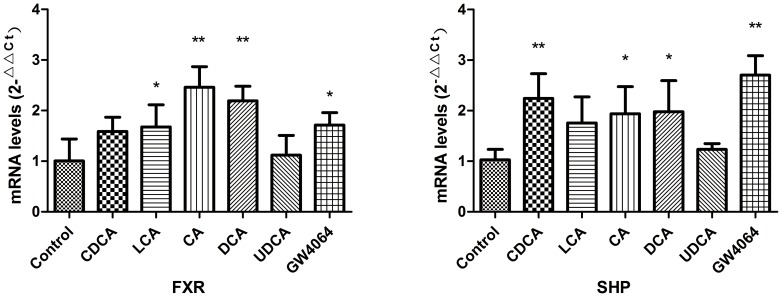
Effects of bile acids and GW4064 on FXR-SHP pathway. (n = 3 independent assays. *P<0.05, **P<0.01 vs. control.)

Bile acids are acknowledged as physiological ligands of FXR. Under our experimental conditions, all four bile acids (CDCA, CA, DCA and LCA) and GW4064 except UDCA were able to increase the mRNA of FXR. As one of the target genes of FXR, SHP was also required in the homeostasis and regulation of bile acids. The mRNA levels of SHP were increased by CDCA, CA, DCA, LCA and GW4064. Similarly, UDCA failed to increase the mRNA of SHP. Although the mRNA levels of FXR increased by CDCA did not reach a significant difference compared to Control, CDCA markedly increased the mRNA of SHP. *See* *Figure 1*.

### 2. Effects of GW4064 on RRDA recorded from hypoglossal nerves

**Figure 2:**
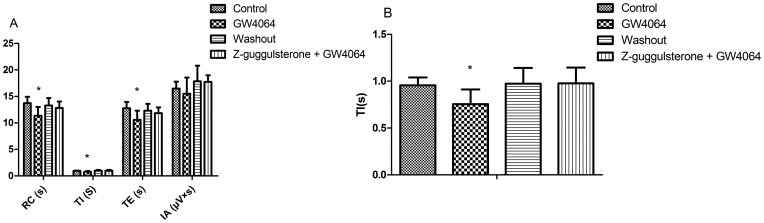
Effects of GW4064 (3 µM) and the FXR blocker Z-guggulsterone (20 µM) on RRDA recorded from hypoglossal nerves (A, B, C). The last figure specifically shows effects on TI(s) of RRDA (D). (n = 6 independent assays. *P<0.05, **P<0.01 vs. control. RC, respiratory cycle; TI, inspiratory time; TE, expiratory time; IA, integral amplitude.)

**Figure 3:**
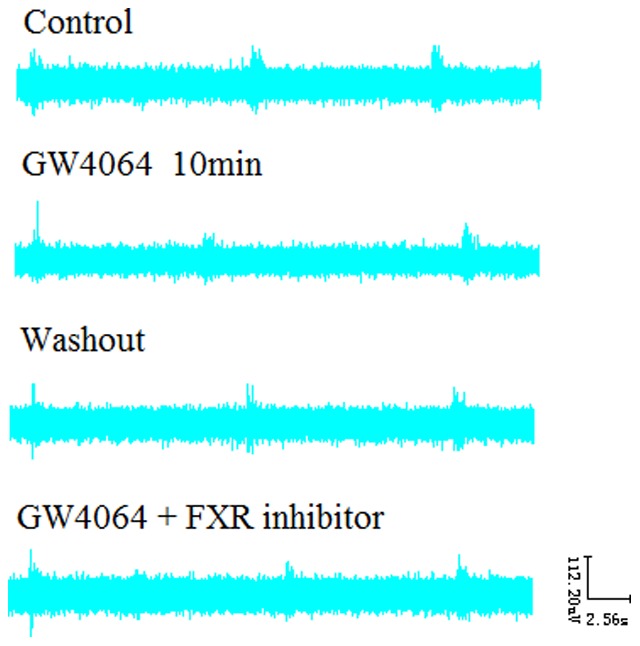
Effects of GW4064 (3 µM) and the FXR blocker Z-guggulsterone (20 µM) on RRDA recorded from hypoglossal nerves in neonatal rat brainstem medulla oblongata slice.

GW4064 is a highly specific synthetic FXR agonist. As our results indicted, GW4064 (3 µM) significantly decreased RC by 17.19%, TI by 20.89% and TE by 16.91%. And such effects were completely reversed by co-administration of GW4064 and Z-guggulsterone (20 µM). *See* *Figure 2*, *3*.

### 3. Effects of CDCA on RRDA recorded from hypoglossal nerves

**Figure 4:**
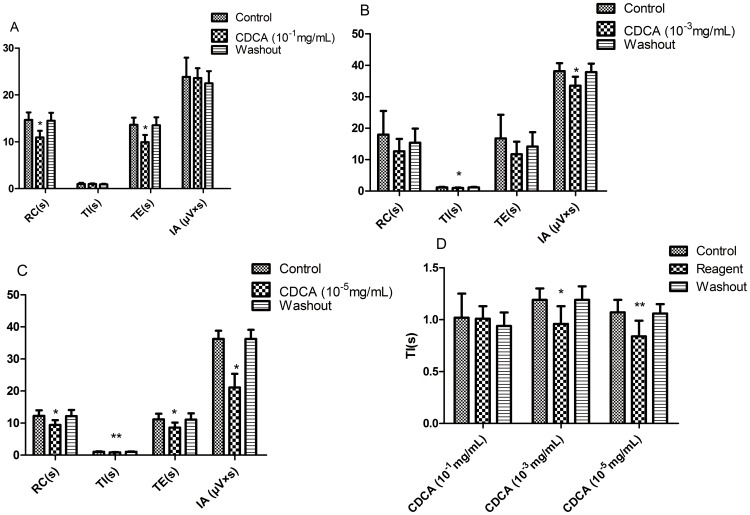
Effects of different concentrations of CDCA (10-1 mg/mL, 10-3 mg/mL, 10-5 mg/mL) on RRDA recorded from hypoglossal nerves (A, B, C). The last figure specifically shows effects of CDCA on TI(s) of RRDA (D). (n = 6 independent assays. *P<0.05, **P<0.01 vs. control. RC, respiratory cycle; TI, inspiratory time; TE, expiratory time; IA, integral amplitude.)

**Figure 5:**
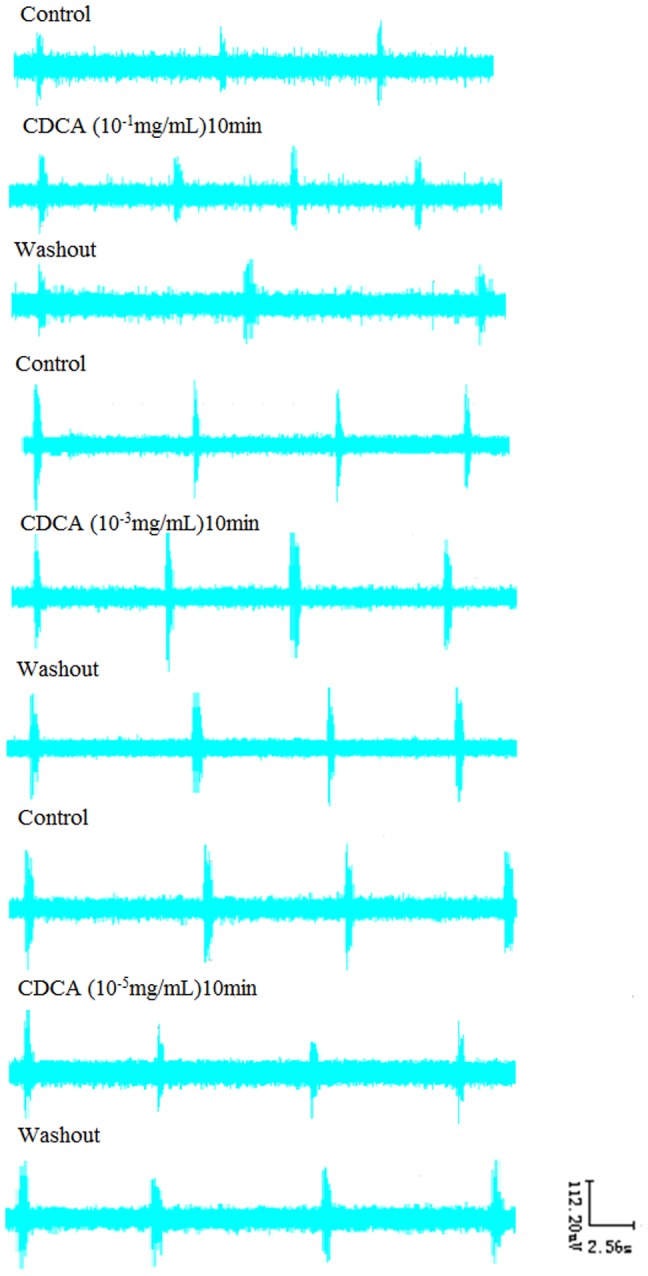
Effects of CDCA on RRDA recorded from hypoglossal nerves in neonatal rat brainstem medulla oblongata slice.

As CDCA is the most potent agonist for FXR, we first investigated the influence of CDCA on RRDA. When applied alone, CDCA at the concentration of 10^−1^ mg/mL decreased RC by 25.37% (*P*<0.05), TE by 27.20% (*P*<0.05). And 10^−3^ mg/mL CDCA decreased TI by 19.33% (*P*<0.05), IA by 12.11% (*P*<0.05). 10^−5^ mg/mL CDCA decreased RC by 22.94% (*P*<0.05), TI by 21.50% (*P*<0.01) and IA by 41.75% (*P*<0.05). *See* *Figure 4*, *5*.

### 4. Effects of DCA on RRDA recorded from hypoglossal nerves

**Figure 6:**
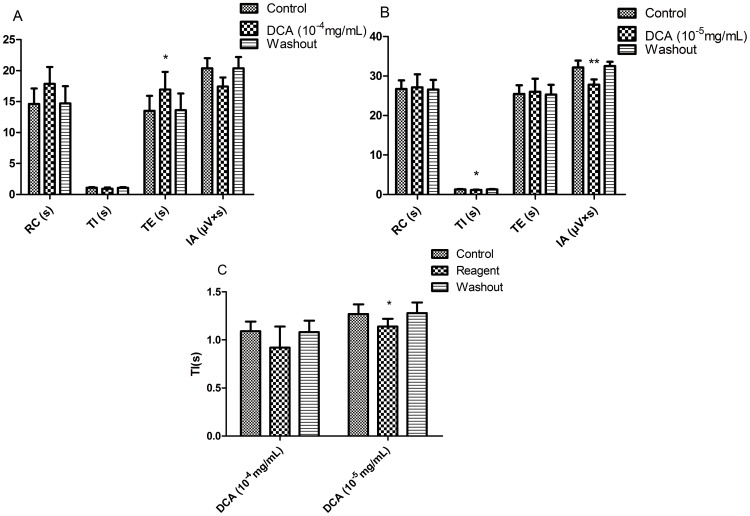
Effects of different concentrations of DCA (10-4 mg/mL, 10-5 mg/mL) on RRDA recorded from hypoglossal nerves (A, B). The last figure specifically shows effects of DCA on TI(s) of RRDA (C). (n = 6 independent assays. *P<0.05, **P<0.01 vs. control. RC, respiratory cycle; TI, inspiratory time; TE, expiratory time; IA, integral amplitude.)

**Figure 7:**
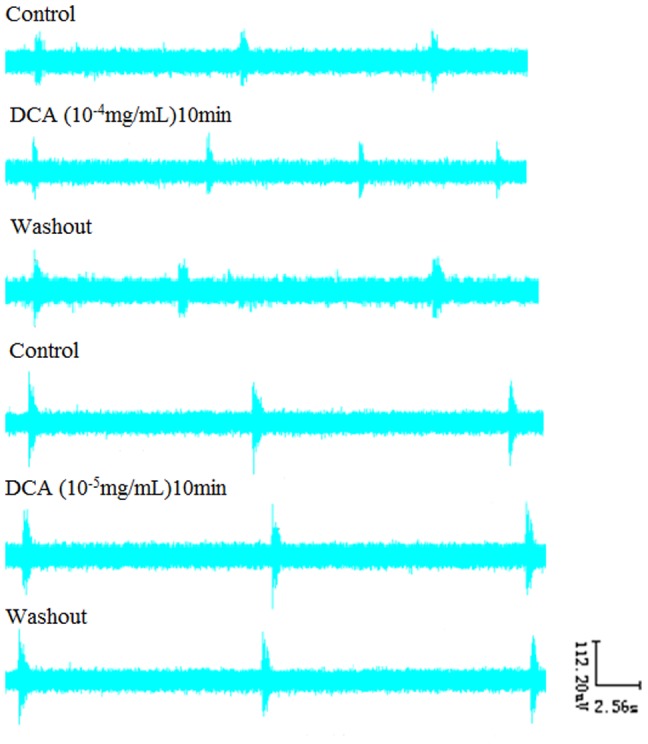
Effects of DCA on RRDA recorded from hypoglossal nerves in neonatal rat brainstem medulla oblongata slice.

We then moved to examine how DCA would affect the RRDA. We found that DCA at the concentration of 10^−4^ mg/mL increased TE by 25.37% (*P*<0.05). And 10^−5^ mg/mL DCA decreased TI by 10.24% (*P*<0.05), IA by 13.61% (*P*<0.05). *See* *Figure 6*, *7*.

### 5. Effects of LCA on RRDA recorded from hypoglossal nerves

**Figure 8:**
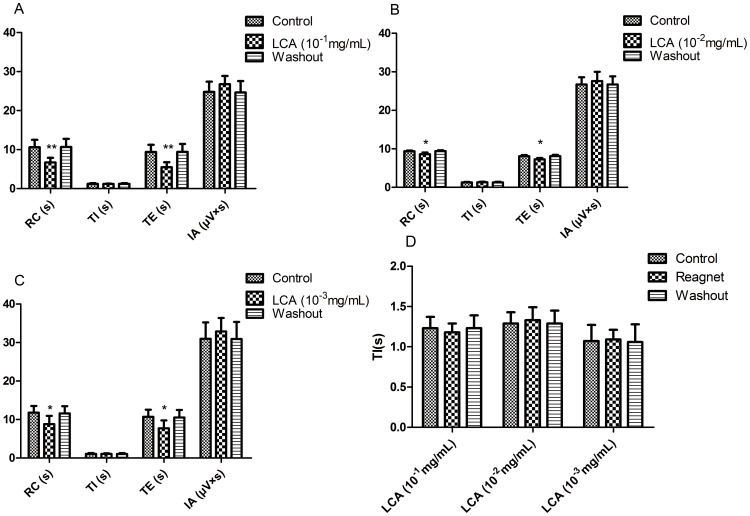
Effects of different concentrations of LCA (10-1 mg/mL, 10-2 mg/mL, 10-3 mg/mL) on RRDA recorded from hypoglossal nerves (A, B, C). The last figure specifically shows effects of LCA on TI(s) of RRDA (D). (n = 6 independent assays. *P<0.05, **P<0.01 vs. control. RC, respiratory cycle; TI, inspiratory time; TE, expiratory time; IA, integral amplitude.)

**Figure 9:**
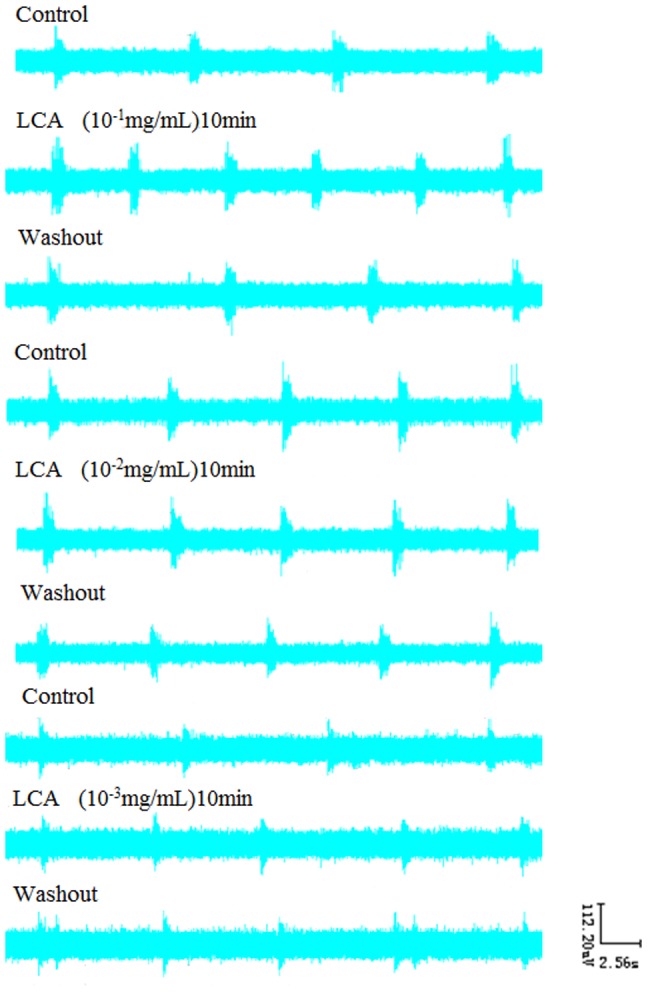
Effects of LCA on RRDA recorded from hypoglossal nerves in neonatal rat brainstem medulla oblongata slice.

After we had a look at how CDCA and DCA influenced the RRDA, we showed that LCA at the concentration of 10^−1^ mg/mL decreased RC by 37.25% (*P*<0.01), TE by 41.60% (*P*<0.01). And 10^−2^ mg/mL LCA decreased RC by 8.22% (*P*<0.05), TE by 10.02% (*P*<0.05). 10^−3^ mg/mL LCA decreased RC by 25.19% (*P*<0.05) and TE by 27.99% (*P*<0.05). *See* *Figure 8*, *9*.

### 6. Effects of CA on RRDA recorded from hypoglossal nerves

**Figure 10:**
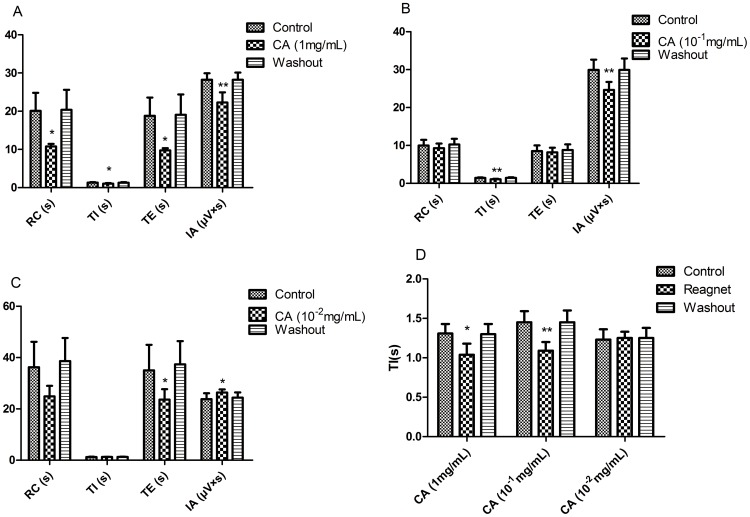
Effects of different concentrations of CA (1 mg/mL, 10-1 mg/mL, 10-2 mg/mL) on RRDA recorded from hypoglossal nerves (A, B, C). The last figure specifically shows effects of CA on TI(s) of RRDA (D). (n = 6 independent assays. *P<0.05, **P<0.01 vs. control. RC, respiratory cycle; TI, inspiratory time; TE, expiratory time; IA, integral amplitude.)

**Figure 11:**
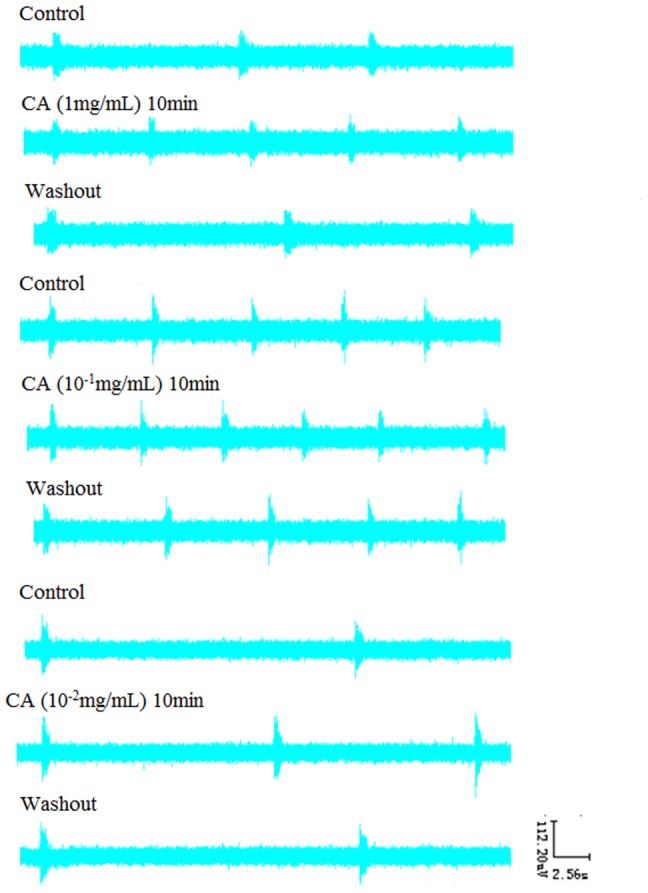
Effects of CA on RRDA recorded from hypoglossal nerves in neonatal rat brainstem medulla oblongata slice.

As one of the known agonists of bile acids for FXR, CA at the concentration of 1 mg/mL decreased RC by 46.31.064% (*P*<0.05), TI by 20.61% (*P*<0.05), TE by 48.14% (*P*<0.05) and IA by 21.14% (*P*<0.01). And 10^−1^ mg/mL CA decreased TI by 24.83% (*P*<0.01), IA by 17.58% (*P*<0.01). 10^−2^ mg/mL CA decreased TE by 32.51% (*P*<0.05) and increased IA by 10.70% (*P*<0.05). *See* *Figure 10*, *11*.

### 7. Effects of UDCA on RRDA recorded from hypoglossal nerves

Under our experimental conditions, UDCA at different concentrations (1∼10^−3^ mg/mL) was separately applied with no significant influences on RC(s)?TI(s)?TE(s)?IA ( µV×s) of RRDA recorded from hypoglossal nerves compared to controls. Thus, data was not illustrated in this article.

### 8. Effects of FXR blocker Z-guggulsterone and Z-guggulsterone with four bile acids on RRDA recorded from hypoglossal nerves

**Figure 12:**
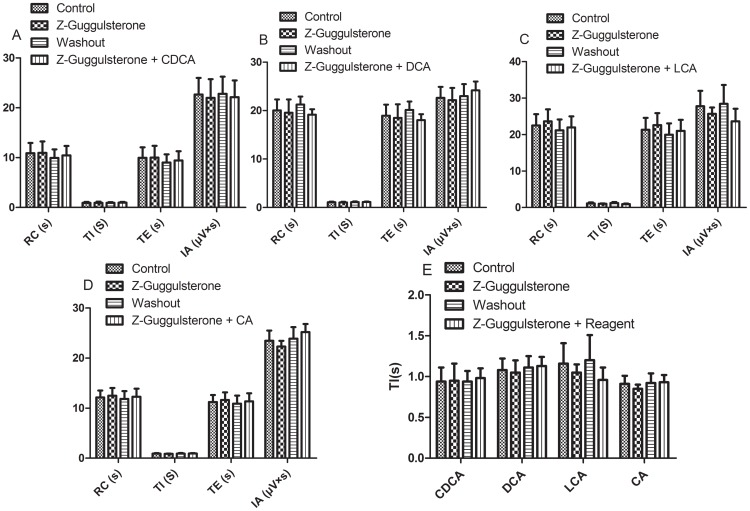
Effects of the FXR blocker Z-guggulsterone (20 µM), as well as the combination of Z-guggulsterone and the four bile acids (CDCA, DCA, LCA, CA) on RRDA recorded from hypoglossal nerves (A, B, C, D). The last figure specifically shows effects of the above reagents on TI(s) of RRDA (E). (n = 6 independent assays. RC, respiratory cycle; TI, inspiratory time; TE, expiratory time; IA, integral amplitude.)

**Figure 13:**
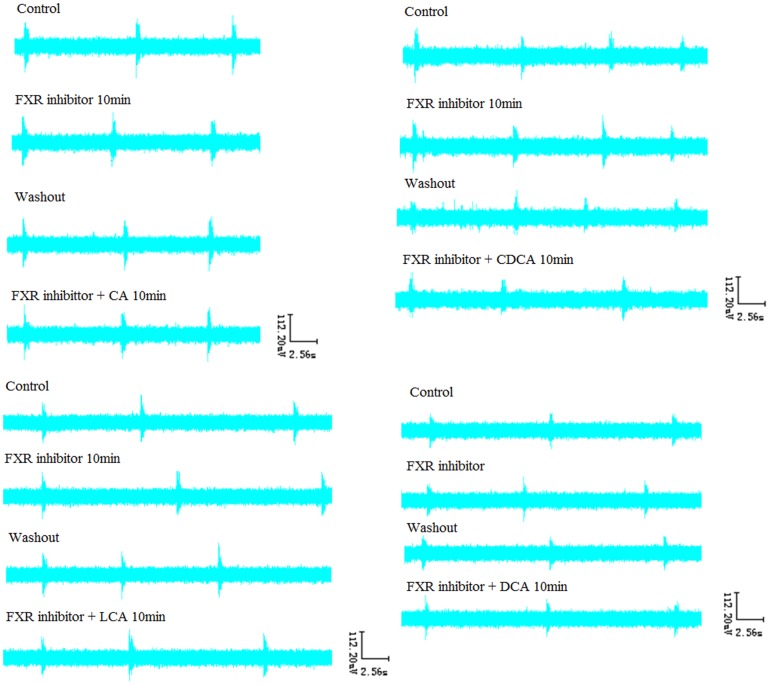
Effects of FXR blocker Z-guggulsterone, as well as Z-guggulsterone and four bile acids on RRDA recorded from hypoglossal nerves in neonatal rat brainstem medulla oblongata slice.

After RRDA was stably recorded from hypoglossal nerves, FXR blocker Z-guggulsterone (20 µM) was applied. As a result, no significant differences compared to the control were observed. Then, Z-guggulsterone was washed out. When full recover of the RRDA was achieved, we used the combination of Z-guggulsterone and the four bile acids. And again, there were no significant differences. *See* *Figure 12*, *13*.

### 9. Effects of DMSO on RRDA recorded from hypoglossal nerves

**Figure 14:**
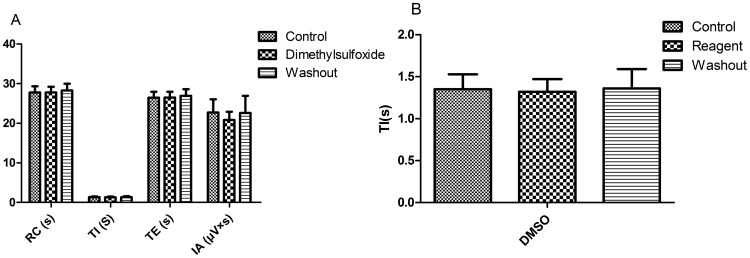
Effects of DMSO on RRDA recorded from hypoglossal nerves (A). The last figure specifically shows effects of DMSO on TI(s) of RRDA (B). (n = 6 independent assays. RC, respiratory cycle; TI, inspiratory time; TE, expiratory time; IA, integral amplitude.)

After RRDA was stably recorded from hypoglossal nerves, DMSO (final concentration was 0.5%) was applied to observe its effects. As a result, no significant differences compared to the control were observed. *See* *Figure 14* *,* *15*.

**Figure 15:**
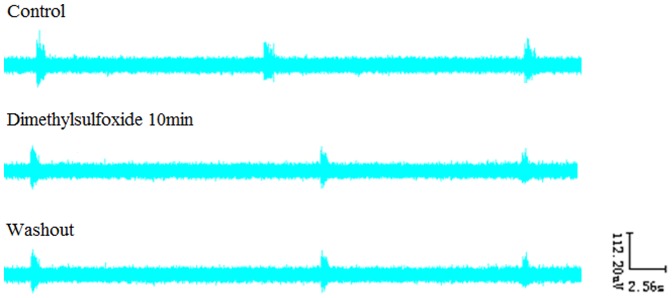
Effects of DMSO on RRDA recorded from hypoglossal nerves in neonatal rat brainstem medulla oblongata slice.

## Discussion

Smith and his co-workers [3] proposed in 1991 that the pre-Bötzinger complex located within the ventrolateral medulla may be responsible for spontaneous respiratory movements. Johnson and his team [25] managed to reduce the thin transverse medullary slice to isolated pre-Bötzinger “islands” and showed that the isolated islands remained rhythmically active and pre-Bötzinger was the kernel for rhythmogenesis in the slice. Periodic respiratory rhythm may be generated out of two different but functionally interacting networks: the retro-trapezoïd/parafacial respiratory group (RTN/pFRG) [26], [27] and the pre-Bötzinger complex (preBötC). As shown in recent studies, the preBötC governs inspiration, and the RTN/pFRG controls active expiration [28]. The *in vitro* preparation of brain slice proves to be useful experimental tools as its spontaneous periodic nerral activity recorded from cranial nerves corresponds to the respiratory rhythm produced in the brain stem of intact animals. Although the periodic rhythm of this *in vitro* preparation has some significant differences compared to normal respiratory rhythm in the intact animal due to reasons such as absence of the afferent inputs from the periphery, the preparation is still considered valuable for physiological and biochemical investigations of the respiratory rhythm generation and modulation in mammalian central nervous system. Thus, we used the *in vitro* brainstem medulla slice of neonatal Sprague-Dawley rats in our study.

According to our previous studies, the decrease of the blood pressure resulted from classical depressor reflex by stimulation of pneumogastric nerves resembled that resulted from intravenous injection of diluted bile acids in terms of the total blood pressure reaction time. The reaction time here we referred to marks the period between onset of the effect and the time point when the effect diminished. However, changes in respiratory rhythm are not the same situation. After stimulation of pneumogastric nerves, the total respiratory reaction time lasted nearly equal to the total blood pressure reaction time. But intravenous injection of diluted bile acids caused a 9–10 fold longer respiratory reaction time, as well as higher peak amplitude. We then analyzed that the significant changes in respiratory functions caused by intravenous injection of diluted bile acids compared to pneumogastric nerves stimulation could be due to the additive effects of bile acids and reflex responses. This indirectly proved that bile acids could be involved in the regulation of respiratory rhythm while no exact elucidation of the underlying mechanism was available. And moreover, injection of UDCA led to no significant changes in blood pressure or respiratory rhythm. Also, the changes in blood pressure caused by intravenous injection of bile acids were not in accordance with those in respiratory rhythm [23].

Based on our experiment, GW4064, as a well acknowledged agonist of FXR, and all of the four bile acids CDCA, LCA, CA, DCA except UDCA exerted significant effects on the spontaneous periodic respiratory-related rhythmical discharge activity (RRDA) recorded from hypoglossal nerves in a concentration-dependent manner, altering changes on four aspects of respiratory cycle (RC), inspiratory time (TI), expiratory time (TE), and integral amplitude (IA) *see* *Figure 2*, *4*, *6*, *8*, *10*. However, when co-administered with the FXR inhibitor Z-guggulsterone, the respiratory changes resulted from GW4064 and the four above bile acids were completely reversed *see* *Figure 12*, *13*. We also showed that each of the four bile acids seemed to have specific influence on the RRDA. DCA mainly affected TE, TI and IA with no changes observed on RC. And also, LCA exerted its effects only through RC and TE. In the meanwhile, CDCA, LCA and CA all had significant effects on RC apart from DCA. Besides the above results, we also confirmed that even for the same bile acid, its effects on the RRDA changed accordingly to its concentration. Besides the influence of LCA on RC and TE at all concentrations, other bile acids at different concentrations had distinct effects. For instance, CA only at the 1 mg/mL seemed to affect RC. Our results indicated here that different kinds of bile acids would have specific effects on respiratory rhythm and different concentrations of the same bile acid could also have distinct influences on respiration. However, an explanation of how these bile acids affected the respiration could not be provided due to our limitations. Of note, all the above effects of bile acids at different concentrations could be reversed by farnesoid X receptor blocker Z-guggulsterone, with no changes observed on RC, TI, TE and IA compared to control groups. So we inferred that bile acids exerted their functions on respiratory rhythm via FXR. Although bile acids tended to affect RRDA mainly by decreasing RC, TE and TI, there was one exception that DCA at the concentration of 10^−4^ mg/mL increased TE by 25.37%. Due to our limitations, possible explanations including the signaling networks and mechanisms on how bile acids exerted their specific effects via FXR in the brain could not be given. For purposes of accurate mechanisms, further studies are required.

Taken all the above together, it is interesting to speculate that the bile acids may affect respiration in two separate ways, the respiratory frequency and the respiratory pattern. And by respiratory pattern we mean that while the respiratory frequency stays the same, the inspiratory and expiratory time change. With RC, TI and TE as our measurement indicators, DCA only affect the respiratory pattern while CDCA, LCA, CA have not only influence on the frequency, but also on both the inspiratory and expiratory time. Almost in all concentrations of CA, LCA and CDCA, the TI and TE tend to decrease in a concentration-dependent manner, resulting in higher respiratory frequencies. This again proves the involvement of bile acids in the regulation of respiration, exerting their effects on respiratory frequency, pattern and amplitude.

As a member of nuclear hormone receptor superfamily, farnesoid X receptor (FXR) plays a pivotal role in maintaining the homeostasis of cholesterol and bile acids. FXR carries out most of its functions by altering the expression of its target genes such as bile salt export pump (BSEP) through binding to the FXR response element (FXRE). The influences of CA, DCA, LCA and CDCA on respiratory rhythm could be reversed by the FXR blocker Z-guggulsterone, indicating the role of FXR in the respiratory regulation. As natural activators of FXR, CDCA, CA, DCA and LCA bind to FXR with decreasing potency [29]. It is possible to speculate that the above four bile acids firstly activate FXR, thereafter allowing the downstream genes of FXR to get involved in the respiratory regulation. However, changes in respiratory function of these four bile acids are not in accordance with their affinity to FXR, suggesting that other potential mechanisms may regulate the respiration. UDCA does not activate FXR, which supports our observation that UDCA exerted no significant effects on respiration [12].

**Figure 16:**
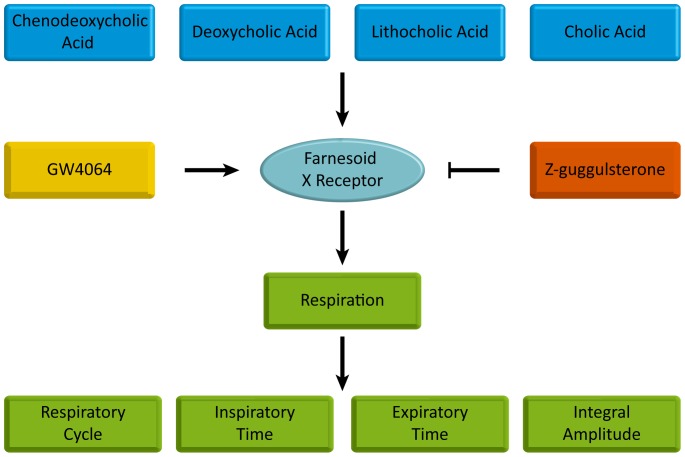
Schema of effects on respiration. CDCA, DCA, LCA, CA and FXR agonist GW4064 influence respiration via FXR represent by changes in respiratory cycle, inspiratory time, expiratory time and integral amplitude. To confirm the involvement of FXR, the FXR inhibitor Z-guggulsterone is co-administered with the above bile acids and GW4064, completely reversing their effects on respiration.

In this experiment, we confirm that CA, DCA, LCA, CDCA and GW4064 are all able to regulate respiratory rhythm except UDCA. And activation of FXR is probably involved in such process as FXR inhibitor Z-guggulsterone completely reversed the effects of the above bile acids and GW4064 *see* *Figure 16*. A novel function of FXR in the brainstem medulla beyond its traditional roles in liver and intestine is confirmed in our research, providing potential therapeutic targets in treating respiratory diseases. However, due to our limitations, no exact pathways or mechanisms are given, which needs further investigations.
